# Inflammatory Targets in Diabetic Nephropathy

**DOI:** 10.3390/jcm9020458

**Published:** 2020-02-07

**Authors:** Javier Donate-Correa, Desirée Luis-Rodríguez, Ernesto Martín-Núñez, Víctor G. Tagua, Carolina Hernández-Carballo, Carla Ferri, Ana Elena Rodríguez-Rodríguez, Carmen Mora-Fernández, Juan F. Navarro-González

**Affiliations:** 1 Unidad de Investigación, Hospital Universitario Nuestra Señora de Candelaria, 38010 Santa Cruz de Tenerife, Spain; jdonatecorrea@gmail.com (J.D.-C.); emarnu87@gmail.com (E.M.-N.); vtagua@funcanis.es (V.G.T.); carlamferri@gmail.com (C.F.); carmenmora.fdez@gmail.com (C.M.-F.); 2 GEENDIAB (Grupo Español para el estudio de la Nefropatía Diabética), Sociedad Española de Nefrología, 39008 Santander, Spain; 3 Servicio de Nefrología, Hospital Universitario Nuestra Señora de Candelaria, 38010 Santa Cruz de Tenerife, Spain; desireeluis@gmail.com; 4 Escuela de Doctorado y Estudios de Posgrado, Universidad de La Laguna, 38200 San Cristóbal de La Laguna, Spain; 5 Servicio de Medicina Interna, Hospital Nuestra Señora de Candelaria, 38010 Santa Cruz de Tenerife, Spain; carolinahdezcarballo@gmail.com; 6 Unidad de Investigación, Hospital Universitario de Canarias, 38008 San Cristóbal de La Laguna, Spain; anarrguez@gmail.com; 7 REDINREN (Red de Investigación Renal-RD16/0009/0022), Instituto de Salud Carlos III, 28029 Madrid, Spain; 8 Instituto de Tecnologías Biomédicas, Universidad de La Laguna, 38010 San Cristóbal de La Laguna, Spain

**Keywords:** renal disease, diabetes, inflammation

## Abstract

One of the most frequent complications in patients with diabetes mellitus is diabetic nephropathy (DN). At present, it constitutes the first cause of end stage renal disease, and the main cause of cardiovascular morbidity and mortality in these patients. Therefore, it is clear that new strategies are required to delay the development and the progression of this pathology. This new approach should look beyond the control of traditional risk factors such as hyperglycemia and hypertension. Currently, inflammation has been recognized as one of the underlying processes involved in the development and progression of kidney disease in the diabetic population. Understanding the cascade of signals and mechanisms that trigger this maladaptive immune response, which eventually leads to the development of DN, is crucial. This knowledge will allow the identification of new targets and facilitate the design of innovative therapeutic strategies. In this review, we focus on the pathogenesis of proinflammatory molecules and mechanisms related to the development and progression of DN, and discuss the potential utility of new strategies based on agents that target inflammation.

## 1. Introduction

Diabetes has become a global health burden affecting 425 million people worldwide according to the International Diabetes Federation (IDF) [1]. IDF also estimates that this number will increase to 630 million in 2045. One of the most important medical complications in diabetic population is the diabetic nephropathy (DN). In the western world, one-third of all diabetic individuals are affected by this pathology [2], which is the leading cause of end-stage renal disease (ESRD) [3,4]. Additionally, renal disease is a major cause of cardiovascular morbidity and mortality in patients with diabetes, with this epidemic being likely to drive us to previously unforeseen socioeconomic costs [5]. Therefore, it is urgent to establish new, effective, and safe therapeutic strategies against DN based on the understanding of the intricate molecular mechanisms of the disease.

The current understanding of the pathogenesis of DN recognizes the involvement of a myriad of deviations from normal homeostasis, including hemodynamic abnormalities, that trigger the increase in systemic and intraglomerular pressure, metabolic abnormalities, oxidative stress, fibrosis, and the activation of the renin–angiotensin system (RAS) [6,7]. Several therapeutic interventions have been implemented to slow the progression of DN. However, these approaches have proved insufficient and new strategies are warranted.

Both circulating inflammatory mediators and infiltration of immune cells levels into renal tissue have been found to be increased in animal models and in patients with DN [8,9,10,11,12,13,14,15,16,17,18]. Adhesion molecules and chemokines are also upregulated in diabetic kidneys, highlighting the role of inflammation in the development of renal damage in the setting of diabetes [18,19,20,21,22,23,24]. The understanding of the inflammatory mechanisms involved in the development and progression of this disease will enable the identification of new potential targets and facilitate the design of innovative anti-inflammatory therapeutic strategies. In this review, we focus on the pathogenesis of the proinflammatory molecules and pathways related to the development and progression of DN, and discuss the opportunity of potential new strategies based on the agents that target the inflammation.

## 2. Inflammation in the Setting of DN

Chronic low-grade inflammation and activation of the innate immune system are key factors in the pathogenesis of diabetes mellitus [8,9,10]. Diverse inflammatory parameters are elevated in diabetic patients [11,12,13,14,15] and constitute strong predictors of the development of this disease [16,17,18]. Interestingly, an increasing number of studies also suggest that inflammation, together with oxidative stress and fibrosis, are key links in the progression of DN [19], in addition to hemodynamic abnormalities, metabolic derangements, and increased synthesis of neurohumoral factors such as angiotensin II.

Diverse components of the immune system participate in the initiation and progression of DN including adhesion molecules, chemokines, and proinflammatory cytokines. Renal tissue of patients with DN revealed increased levels of inflammatory molecules and the accumulation of high levels of inflammatory cells [20,21,22,23,24]. The concentration of these components increase as nephropathy progresses [19,25], and all of which have been related to increased urinary albumin excretion (UAE) and to clinical markers of glomerular and tubulointerstitial damage [25,26].

### 2.1. Inflammatory Cell Accumulation in the Kidney

Immunologic mechanisms play a significant role in development and progression of DN [27], with recruitment and activation of innate immune cells and elaboration of proinflammatory molecules. The expression of chemokines and adhesion molecules is upregulated in renal cells of animal models of diabetes and in patients with diabetes. These molecules are key mediators of renal injury by virtue of their ability to attract circulating white blood cells and facilitate transmigration of these cells into renal tissue. Adhesion molecules include the vascular cell adhesion molecule 1 (VCAM1) and the intercellular adhesion molecule 1 (ICAM1). Both play an important role in the initiation of renal inflammation. These molecules have been abundantly found in renal biopsies obtained from patients with DN, and circulating levels of both have been related to DN progression [28,29]. In mice models, the deletion of the ICAM1 gene ameliorates renal inflammation [30], indicating that ICAM1 contributes to the pathogenesis of DN.

The renal accumulation of monocytes and macrophages has been related to the progression of chronic kidney disease (CKD) [31]. Thereby, this accumulation has been associated with a decline in the glomerular filtration rate (GFR), the manifestation of histological changes, and poor outcomes in DN patients [20,21,22,23,24,31,32]. In addition, infiltrating cells in the renal microenvironment constitute a source of reactive oxygen species (ROS), proinflammatory factors, metalloproteinases, and growth factors, all of which amplify and chronify the inflammatory response and the progression of kidney damage [33].

The inhibition of this inflammatory cell recruitment has been shown to be protective in experimental diabetic nephropathy [34,35]. Mice models with a deletion in macrophage receptors are more resistant to the development of DN through the amelioration of inflammation and the reduction in the levels of albuminuria, mesangial matrix expansion, and transforming growth factor (TGF-) [36].

In a physiologic inflammatory reaction, macrophages linearly progress through the M1 and M2 phases. M1 macrophages initiate the immune response and produce proinflammatory cytokines. Conversely, M2 are responsible of the tissue repair producing either polyamines to induce proliferation or proline to induce collagen. Under chronic inflammatory conditions, this order of events disappears and both forms coexist. Notably, the M1 phenotype is the major macrophage in streptozotocin-induced DN animals [32,37]. Interestingly, the switch from the M1 to the M2 state induced by the deletion of the Toll-like receptor-2, attenuates UAE and the development of renal morphological changes [37]. The mechanisms by which M2 macrophages attenuate the progression of DN and promote kidney repair are unknown.

### 2.2. Chemokines, Cytokines, and Signaling Pathways Related with Inflammation

**Figure 1:**
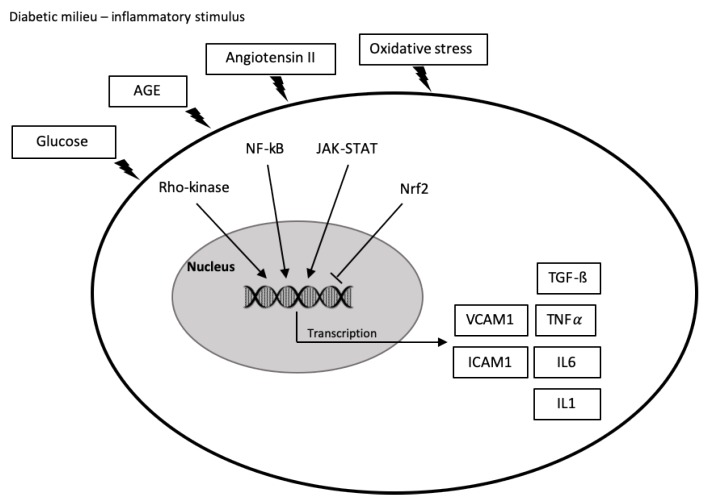
Overview of the participation of inflammatory mechanisms in the pathophysiology of diabetic nephropathy. In the diabetic milieu, glucose, advanced glycation end products (AGEs), angiotensin II, and oxidative stress activate a variety of signaling cascades leading the production of chemokines and cytokines that drive monocyte infiltration and the development of inflammation. AGE, advanced glycation end product; nuclear transcription factor kappa B (NFκB); JAK-STAT, Janus kinase/signal transducer and activator of transcription; Nrf2, nuclear factor-2 erythroid related factor 2; VCAM1, vascular cell adhesion molecule 1; ICAM1, intercellular adhesion molecule; TGF-β, transforming growth factor beta; TNF, tumor necrosis factor alpha; IL, interleukin.

Together with inflammatory cells, diverse molecules and pathways participate in the development and progression of both systemic and local inflammation in DN. Cytokines and chemokines constitute a group of secreted proteins that regulate much of the immune response. In addition to this role, these molecules exert important pleiotropic actions as cardinal effectors of injury [38]. Moreover, growing evidence supports the role of different metabolic pathways and factors in the activation of inflammatory mechanisms involved in the pathophysiology of DN including the Janus kinase/signal transducers and activators of transcription (JAK/STAT) pathway, the nuclear transcription factor kappa B (NF-κB), the Rho-Kinase Signaling, and the nuclear factor erythroid 2-related factor 2 (Nrf2) (Figure 1).

#### 2.2.1. Chemokines

MCP1—The chemokine monocyte chemoattractant protein 1 (MCP1) is responsible of the migration of the monocytes through the endothelium after the adhesion and is a major factor influencing macrophage accumulation in renal disease patients and in animal models of renal damage [38]. In diabetic patients, MCP1 is upregulated in the glomerular and renal tubular epithelium [39,40]; moreover, urinary levels are strongly associated with the decline of renal function [41]. The expression of MCP1 in renal cells is induced by inflammatory cytokines and constitutes the starting point for the development of glomerular and tubular inflammation. A number of cytokines are involved in the induction of MCP1 expression in the kidney, but several studies point to tumor necrosis factor (TNF) as the most potent inducer. Based on the above data, the MCP1 protein has been proposed as a novel biomarker of tubulointerstitial changes and as predictor of renal progression and prognosis in patients with diabetes [42].

CSF1—The chemokine colony-stimulating factor 1 (CSF1) governs the survival, proliferation, and differentiation of macrophages. Although CSF1 is constitutively expressed in diverse renal cellular types (glomerular mesangial, tubular, and endothelial cells), the disarrangement in the renal levels of this chemokine in chronic inflammation is involved in the progression of renal disease by generating an uncontrolled intrarenal amplification of macrophages. Renal CSF1 is highly expressed in type 2 diabetic *db/db* mice [43]. Interestingly, decreasing the levels of renal CSF1, by deletion of the codifying gene in mice, attenuates the infiltration and proliferation of macrophages during renal inflammation [44]. The accumulation of macrophages is also attenuated in diverse animal models of renal diseases by the administration of an antibody against the receptor of CSF1 [45]. The regulation of the levels of this factor raises the possibility of specific therapies for targeting macrophage-mediated injury in DN.

#### 2.2.2. Inflammatory Cytokines

TNF—The cytokine TNF is mainly produced by monocytes but also, in a lower extent, by renal cells (endothelial, epithelial, mesangial, and tubular cells) [46,47,48]. TNF plays a significant role in the progression of renal disorders [48] interacting with the kidney in a paracrine or autocrine manner [48,49,50,51]. Serum and urinary levels of TNF are increased in patients with DN when compared with nondiabetic individuals or with diabetic subjects without renal disease. Importantly, the increase in TNF levels is related with the development and progression of renal disease in patients with diabetes [52,53]. The expression levels of TNF protein are also enhanced in renal glomeruli and tubules of animal models of diabetes [53,54,55,56,57]. Hypertrophy and hyperfiltration are prominent signs in DN and both have been related to increased TNF expression levels. Several harmful effects are elicited in the kidney by TNF including cytotoxicity [58], apoptosis, and necrotic cell death [59,60]. TNF also alters the equilibrium between vasoconstriction and vasodilation, changing the permeability of endothelial cells, which leads to alterations of intraglomerular blood flow and reductions in GFR [61]. Moreover, TNF directly prompts the formation of ROS in the renal cells independently of hemodynamic mechanisms, altering the glomerular capillary wall and consequently, increasing UAE [62,63,64]. Finally, TNF stimulates sodium retention that might induce the expression of TFG- and consequently, the development of renal hypertrophy [65,66]. These harmful effects can be abrogated by a soluble TNF receptor fusion protein, by blockers of the renal epithelial sodium channel, such as amiloride, and by inhibitors of extracellular signal-related protein kinase.

IL6—The levels of the cytokine interleukin (IL) 6 are also increased in patients with DN when comparing to diabetic patients without renal disease [67]. Kidneys of patients with DN also present increased expression levels of IL6 in infiltrating cells from the mesangium, interstitium, and tubules. Diverse abnormalities at kidney level have been associated with the raise in the expression of IL6 including changes in the permeability of glomerular endothelium, expansion of the mesangium, increased fibronectin levels [68,69], and thickness of the glomerular basement membrane [70,71]. Renal cells of diabetic kidneys also express increased levels of IL6 and experimental studies in animal models with diabetes positively associated this expression with the urinary concentration of this cytokine and with the development of renal hypertrophy [54,55].

IL18—Serum and urinary levels of the cytokine IL18 are increased in DN, being significantly correlated with UAE levels [52,72,73]. IL18 is a potent proinflammatory cytokine with pleiotropic functions including the synthesis of diverse molecules involved in the inflammatory process like IL1 and TNF, the release of interferon- (IFN-) [74], which stimulates functional chemokine receptor expression in mesangial cells [75], the increase in the expression of ICAM1, and the apoptotic process in endothelial cells [76,77,78]. Renal tubular cells express increased levels of IL18 in patients with DN [79], which has been related to the triggering of mitogen-activated protein kinase (MAPK) pathways secondary to the action of TGF- [80]. Moreover, infiltrating cells in the renal tissue also produce this cytokine [81,82].

#### 2.2.3. Inflammatory Signal Transduction

Several signaling cascades play a critical role in the development and progression of renal inflammation. These pathways transduce the signals of diverse upstream mediators including oxidative stress, angiotensin II, and advanced glycation end products (AGEs), resulting in a decline in renal function. 

JAK/STAT pathway—The JAK proteins are a family of intracellular, non-receptor, tyrosine kinases that transduce extracellular signals after binding to the membrane receptors. The JAK proteins associated with the intracellular domain of the receptor are then phosphorylated and activated. JAK/STAT family is comprised of four tyrosine kinases (JAK1-3 and TYK2) and seven transcription factors (STAT1-4, 5a, 5b and 6), with particular cell-specific combinations identified for each receptor type [83]. The phosphorylated JAK proteins suffer a conformational modification, allowing the transduction of the intracellular signal by further phosphorylating and activating the STAT transcription factors. The activated STAT molecules translocate to the cell nucleus, where they activate many target genes. The JAK/STAT pathway plays a critical role in diverse renal cell types, transducing diverse signals from extracellular ligands including cytokines, chemokines, growth factors, and hormones [84]. The activation of JAK/STAT is an important mechanism by which hyperglycemia contributes to renal damage participating in the pathogenesis of DN through its participation in several processes, such as the hypertrophy of mesangial cells induced by angiotensin Ⅱ, and the synthesis of TGF-, collagen Ⅳ, and fibronectin. In animal models of DN, it has been demonstrated that hyperglycemia is able to turning on the JAK/STAT pathway in the glomeruli and tubulointerstitial cells [85,86,87,88,89,90]. Similarly, gene and protein expression studies of kidney biopsies from patients with early or advanced stages of DN have shown increased activation and expression of the of JAK/STAT [89,91]. In these patients, the increment in mRNAs levels of many JAK/STAT components in the glomerular and tubulointerstitial compartment was inversely correlated with the estimated GFR [89]. Moreover, gene expression and activity of JAK1 and JAK2 have been related to the progression of DN [84].

The proposed mechanism by which hyperglycemia activates the JAK/STAT pathway is through the activation of the activity of JAK proteins caused by ROS under high glucose conditions. Diphenylene iodonium, an inhibitor of ROS formation, results in a marked inhibition of angiotensin Ⅱ (Ang Ⅱ)-induced activation of JAK proteins in human cardiomyocytes [90]. These results reveal that oxidative stress acts as an intracellular activator of the JAK/STAT pathway, and that ROS also acts as a second messenger for the regulation of JAK proteins activation by Ang Ⅱ. Hyperglycemia also increases JAK proteins tyrosine phosphorylation by the alteration of tyrosine phosphatases (SHP-1 and SHP-2) activity. SHP-1 phosphorylation is abolished and SHP-2 phosphorylation is increased under hyperglycemia, suggesting that JAK sustained activation under hyperglycemia is partly due to decreased SHP-1 and increased SHP-2 phosphorylation [85,86,87,88,89,90].

The tyrosine kinase inhibitor of JAK proteins AG490 is able to abrogate the elevation of systolic blood pressure and the increase of UAE [92,93] in animal models of DN. The proteins suppressors of cytokine signaling (SOCS), a group of molecules that bind and interfere with initiating JAK proteins, are important regulators of JAK/STAT activation in DN. Overexpression of SOCS in human mesangial and tubular cells under high concentrations of glucose reversed the glucose-induced activation of this pathway, the expression of STAT-dependent genes and cell proliferation [94]. Similarly, the inoculation of recombinant SOCS1 and SOCS3 adenovirus to diabetic rats improved renal function and reduced mesangial expansion, fibrosis and macrophages renal infiltration. However, further research into JAK inhibitors, SOCS expression or SOCS mimetics is required, given the critical immunomodulatory role of this pathway, with possible adverse effects.

NF-kB—NF-kB constitutes a family of transcription factors that regulates the expression of genes involved in different processes, such as the immune response, cell differentiation and development, apoptosis, cycle progression, inflammation, and tumorigenesis. This pathway is considered one of the major inflammatory pathways involved in DN. NF-κB is continuously present in cells in an inactive state forming a complex with inhibitors of NF-κB (IκBs). Cell surface receptors such as toll-like receptors (TLRs), respond to extracellular stimuli, including hyperglycemia, AGEs, mechanical stress, oxygen radicals, cytokines, angiotensin II, and albuminuria/proteinuria [95,96,97,98]. Many of this signaling molecules that produce the activation of NF-κB are potential targets for the inhibition of this factor and the subsequent inflammatory response. Upon receptors stimulation, IκB-kinases are activated leading to phosphorylation of IκBs which results in polyubiquitination, a sign for destruction of the IκB by proteasome. After this, activated NF-κB enters the nucleus and stimulates the transcription of adhesion molecules, chemokines, inflammatory cytokines, and other molecules related to inflammation and proliferation, all of which are involved in the progression of DN [96]. The activation of NF-κB has been observed in cells of the proximal tubule [97,98] and in renal cortical tissue of experimental models of DN [43]. Proteinuria also stimulates the activation of NF-κB, constituting an important pro-inflammatory stimulus in renal tubular cells [97]. The expression of chemoattractant and adhesive molecules via activation of NF-κB-dependent pathways is also upregulated by excess ultra-filtered protein load in proximal tubular cells [99]. 

The blockade of RAS in diabetic rats provides renoprotective anti-inflamatory effects through the suppression of NF-κB-dependent pathways, beyond the control of blood pressure and proteinuria [100]. Other drugs such as thiazolidinedione, a peroxisome proliferator activated receptor- (PPAR-), have been also associated to a suppressive effect on the activation of NF-κB [101,102], which was related with an attenuation in renal injury also in diabetic rats. In addition, recent experimental studies indicate that suppression of NF-κB activation by various agents, such as 1,25-dihydroxyvitamin D3 [103], cilostazol [104], and curcumin [105], could lead to amelioration of DKD, suggesting the importance of NF-κB as a therapeutic target of DKD.

Rho-Kinase Signaling—The Rho-associated coiled-coil containing protein kinase (Rho-kinase) is a serine-threonine kinase involved in the regulation and cell proliferation, contraction, and migration of cells by acting on the cytoskeleton. The activation of the Rho-kinase signaling have been associated with renal and cardiovascular diseases. Importantly, the activation of this pathway increases MCP1 levels and the chemotaxis of monocytes toward glomerular cells. Moreover, Rho-kinase is also an important regulator of CSF1 production. In mouse models of DN, the experimental inhibition of this pathway reduces UAE, glomerular expansion [106,107], and the infiltration of macrophages [41]. Similar to the blockade of the JAK/STAT pathway, the inhibition of the Rho-kinase signaling constitutes a new therapeutic anti-inflammatory intervention through reduction of macrophage accumulation in the kidneys.

Experimental studies suggest the existence of a mechanistic linkage between Rho-kinase and NF-κB signaling. Rho-kinase is involved in the endothelial activation of NF-κB by thrombin [108], and lysophosphatidic acid [109], and by neuropeptide in colonic epithelial cells [110]. In experimental mice model of renal failure by lipopolysaccharide injection, Rho kinase inhibition attenuated kidney injury in part by attenuation of NF-κB signaling [111]. All these findings point to Rho-kinase as a new therapeutic target against renal inflammation through a reduction in NF-κB activation.

Nrf2—The transcription factor nuclear factor erythroid 2-related factor 2 (Nrf2) is one of the most important regulators of oxidative stress. Nrf2 regulates the expression of antioxidant cytoprotective genes that attenuate systemic oxidative overload. Moreover, activation of Nrf2 reduces renal inflammation by suppressing macrophage inflammatory response by blocking the transcription of pro-inflammatory cytokines including IL1 and IL6 [112]. In experimental mice models of diabetes, the activation of Nrf2 provides atheroprotection through reducing cytokine production and M1 macrophage accumulation [113]. Furthermore, activated Nrf2 exerts protective effects against pathological changes in the glomerulus and attenuates mesangial hypertrophy induced by high glucose [114].

## 3. New (and Old) Therapies Targeting Inflammation in DN

Tight glycemic control is the primary strategy for the prevention of the development of diabetic complications, and micro- and macrovascular complications [115]. The current practice for the treatment of the established DN is based on two pillars: the metabolic regulation and the blood pressure control, with the RAS blockade as the cornerstone therapy [116,117]. This blockade is therapeutically achieved using RAS blockers such as angiotensin converting enzyme inhibitors (ACEi) and angiotensin II receptor blockers (ARBs). This approach effectively slows the progression of the nephropathy both in diabetic and nondiabetic patients. Unfortunately, this approach rarely stops the progression towards the ESRD. Furthermore, the combination of RAS blockers has not been shown to be more effective against the progression of renal disease.; on the contrary, it has been associated with an increase in adverse events. [115,116,117,118,119]. Therefore, new approaches are necessary to delay the development and the progression of the DN, to improve kidney function and to overcome the setbacks with recent trials designed to find effective renoprotection in diabetic patients [119,120,121,122,123]. In this sense, new antidiabetic drugs or old drugs with new uses outside the scope of the original indication have appeared as promising candidates showing beneficial renal effects in DN patients (Table 1).

Although the main therapeutic strategy against the development of DN remains directed toward optimizing the control of the major risk factors including hypertension, hyperglycemia, and dyslipidemia [149], recent studies indicate that most successful strategies must also contain immune-targeting properties that limit the inflammation [150]. Interestingly, several renoprotective treatments currently used in patients with DN have anti-inflammatory effects. Therapeutic RAS blockade reduce proteinuria and effectively slow the progression of diabetic and nondiabetic nephropathies by hemodynamic/antihypertensive but also by anti-inflammatory/antifibrotic actions. The reduction in the inflammatory process is mediated by the inhibition of NF-𝜅B dependent pathways [124], a transcription regulator protein complex that regulate inflammatory signals [151]. Downstream targets of NF- B include adhesion molecules and pro-inflammatory cytokines, all of them related with the development of DN: IL6, TNF𝛼, MCP1, and RANTES (Regulated on Activation, Normal T Cell Expressed and Secreted). Methyl bardoxolone selectively reduces the DNA binding of NF-𝜅B by activating powerful Nrf2-dependent phase 2 inducers, thereby inhibiting the inflammatory reaction [125]. Treatment with this compound improved renal function in DN patients in the short- and long-term in a dose-related manner [152] but, unfortunately, phase III clinical trial yielded serious adverse events and hence, the trial was stopped.

The anti-inflammatory effect elicited by the RAS blockade could be related to the therapeutic efficacy of the RAS inhibitors used in the treatment of DN [153,154,155,156]. However, new agents that specifically target the inflammatory phenomena offers new therapeutic opportunities. The manipulation of the pro-inflammatory chemokine MCP1 signaling axis constitutes one of these opportunities. The administration of the MCP1 antagonizing L-RNA aptamer (Spiegelmer^®^) to uninephrectomized db/db mice, significantly reduced glomerular macrophages infiltration while improved diffuse glomerulosclerosis and inhibited the decline in glomerular filtration rate [157]. The blockade of the MCP1 receptor with other compounds produced similar results in the db/db model [158,159,160]. These results have substantiated the hypothesis that blocking the actions of MCP1 might constitute a new therapeutic anti-inflammatory target in DN. At present time, a phase 2, placebo-controlled clinical trial designed to evaluate the safety and tolerability as well as the renoprotective potential of the MCP1 antagonist emanticap pegol in DN, is underway [126]. Emanticap pegol specifically binds and inhibits MCP1. Treatment was able to decrease UAE respect to baseline even after cessation of the intervention, without differences from the placebo group [126].

Otherwise, the inhibition of the JAK/STAT pathway, which is an important regulator for transducing signals from cytokines and chemokines in renal cells [85] is another anti-inflammatory target to reduce glomerular hypertrophy and the decline in the GFR [161]. Diverse drugs and compounds under investigation have shown anti-inflammatory effects via inhibition of the JAK/STAT pathway in experimental DN [162,163]. The Chinese herbal granule Tangshen Formula improves proteinuria and eGFR in patients with type 2 diabetes mellitus [164]. Results of a Phase 2 multicenter, randomized, multi-dose, placebo-controlled, clinical trial (NCT01683409) demonstrated that the oral drug Baracitinib, which selectively inhibits JAK1 and JAK2, attenuated UAE in a dose dependent manner in patients with DN with residual macroalbuminuria on RAS blockade, demonstrating its potential utility in the treatment of DN [85]. A total of 129 participants were randomly selected to receive placebo or different doses of Barcitinib (0.75, 1.5, or 4 mg) for 24 weeks, followed by a 4 weeks wash-out period. Baricitinib (4 mg/day) significantly reduced albuminuria at weeks 12 and 24 and after 4 weeks of washout compared with placebo. Importantly, Baricitinib also decreased the blood levels of the inflammatory biomarkers ICAM1, TNFR 1 and 2, serum amyloid A, and the urine levels of MCP1 [84]. In addition to the suppression of specific signaling pathways related with inflammation, a few novel anti-diabetic drugs have aroused great interest from scientific researchers. These drugs have been demonstrated to improve albuminuria and other traits of DN in type 1 and 2 diabetes and to exert anti-inflammatory actions that potentially could delay the progression of DN. The group of sodium-glucose cotransporter-2 (SGLT2) inhibitors are promising hypoglycemic agents that have the added advantage of not promoting hyperinsulinemia, weight gain, or hypoglycemia, unlike traditional antidiabetic agents [165]. Their mode of action lies in the effective blockade of glucose reabsorption by SGLT2 at the proximal tubule; thereby, causing glucosuria and reducing blood glucose levels, independently of the insulin action [166]. Beyond glycemic control, secondary outcome analyses of these drugs in cardiovascular safety trials have shown potent renoprotective effects. In particular, slower progression of kidney disease and lower rates of clinically relevant renal events have been reported for empagliflozin in the prospective empagliflozin, cardiovascular outcomes and mortality in type 2 diabetes (EMPA-REG OUTCOME) trial [167] comparing the administration of SGLT2 inhibitor with placebo. Similar results were observed, two years later, in the canagliflozin cardiovascular assessment study (CANVAS) [168]. The results of the dedicated renal trial canagliflozin and renal end points in diabetes with established nephropathy clinical evaluation (CREDENCE) have recently confirmed the effects of SGLT2 inhibitors on the decline of kidney function [169]. Although only few studies have focused on the underlying mechanisms explaining the cardiovascular and renal protection exerted by these drugs [170,171,172], SGLT2 inhibitors are thought to potentially target inflammation [173]. In this sense, the administration of canakinumab to high-risk population directly confers cardiovascular benefits and reduces inflammation by targeting the interleukin-1-beta (IL-1) innate immunity. These results were recently reported in the canakinumab anti-inflammatory thrombosis outcome study (CANTOS) [127]. A secondary analysis of CANTOS also reported that the reduction of high-sensitivity C-reactive protein (hsCRP) is a good predictor of beneficial effects on cardiovascular outcomes [128].

A few smaller pilot studies have also pointed to a reduction of inflammatory markers in patients with type 2 diabetes after the administration of SGLT2 inhibitors. Reductions in serum levels of hsCRP, TNF, IL6, and interferon-γ (IFN-γ) have been observed after administration of canagliflozin, dapagliflozin, and empagliflozin [129,130,131,132,133,134,135]. However, most of these reductions did not reach statistical significance, probably because of the limited number of patients. Solid data of short- and mainly long-term SGLT2 inhibitors anti-inflammatory actions in DN, are still needed. Meanwhile, experimental studies point to the existence of this immunomodulatory effect in DN mediated by the reduction in glucose transport into proximal tubular cells and the subsequent appearing of glucotoxicity, oxidative stress, and inflammation, but also by controlling the gene expression of inflammatory molecules by unknow mechanisms. Administration of and SGLT2 inhibitor reduces albuminuria and tubulointerstitial injury in obese diabetic mice [174,175,176]. In HK2 cells (human kidney proximal tubule cell line), empagliflozin attenuates high glucose-induced expression of Toll-like receptor-4, binding of nuclear deoxyribonucleic acid to NF-κB, and secretion of collagen IV and IL6 [177]. In the atherosclerosis mice model ApoE -/-, empagliflozin reduces the expression of inflammatory mediators such as TNFα, IL1β, and IL6 as well as the infiltration of cells into atheromatous plaques [178]. In animal models with type 1 diabetes, empagliflozin treatment achieved a reduction in NF-κB, MCP1, and IL6 renal levels that was related with the reduction in albuminuria [179]. In type 2 diabetes mice models, the inhibition of SGLT2 also reduced glomerular macrophage infiltration and glomerular sclerosis [180] and attenuated the overexpression of NOX4, TGF-β, osteopontin, and MCP1 in the tubular cells induced by high glucose [181].

The other two groups of anti-diabetic drugs are the dipeptidyl peptidase-4 (DPP-4) inhibitors and the glucagon-like peptide-1 (GLP-1) receptor agonists (GLP-1RA). Several clinical trials suggest that these drugs possess the ability to improve renal function, possibly independently of the glycemic control [182,183]. The renoprotection derived from the treatment with GLP-1RA and DPP-4 have been demonstrated in few experimental studies in animal models with DN. For instance, the DPP-4 inhibitor linagliptin exerts anti-inflammatory effects in the endothelium independently of the glycemic control [136,137]. Diabetic patients treated with vildagliptin or sitagliptin presented a reduction in the levels of markers of inflammation [138]. The group of GLP-1RA also exerts anti-inflammatory renoprotective actions. Exenatide treatment reduced albuminuria and macrophage infiltration in db/db mice (a model of type 2 diabetes) [139] and attenuated albuminuria, mesangial expansion, and reduced the levels of inflammatory cytokines and adhesion molecules in type 1 diabetic rats [140]. Recently, it has been demonstrated that sitagliptin and linagliptin can suppress the activity of NLRP3/inflammasome in an experimental model of renal injury [141]. Potential anti-inflammatory properties for incretin modulators are also being investigated in clinical studies. In the clinical setting, type 2 diabetic patients treated with exenatide or dulaglutide presented reduced levels of CRP [142]. The differential effects of diabetes therapy on blood and renal inflammation are being evaluated in several ongoing trials: GLP-1R agonist (exenatide or liraglutide) vs. a DPP4 inhibitor (NCT02150707) and liraglutide (LIRALBU; NCT02545738 and NCT01847313). The results of these studies will shed light on the anti-inflammatory effects of these treatments.

Pentoxifylline (PTX) (3,7-dimethyl-1-(5-oxohexyl)-3,7-dihydro-1*H*-purine-2,6-dione) is a methyl-xanthine derivative with hemorheological actions that has been clinically used for the treatment of claudication for more than 30 years [184]. Importantly, this drug also presents anti-inflammatory properties that supports its potential application in the renoprotection of the diabetic patient. PTX aroused an early interest as a therapeutic agent in kidney disease due to its hemorheological properties and its potential to decrease intraglomerular pressure [185,186]. Both clinical and experimental studies support antiproteinuric effect of PTX. Recently, the antiproteinuric property of PTX has been associated with anti-inflammatory capacities [187,188,189,190,191,192,193].

PTX has a considerable modulating effect on several proinflammatory cytokines, which includes TNFα [153,187], IL1, IL6, interferon γ [53,54,194], and other molecules also related with the inflammatory phenomena like ICAM1, VCAM1, and CRP [195,196]. Most of the clinical trials conducted to assess the renal effect of PTX in the diabetic setting have evidenced the protective effects of this compound by decreasing proteinuria and, in some cases, improving GFR [185,197,198,199,200]. Importantly, in some of these studies [197,201] the antiproteinuric effect of PTX was associated with significant reductions in TNFα levels. Similarly, clinical trials conducted in CKD patients with stage 3 or higher reported a stabilization of renal function and decreased circulating levels of TNFα, fibrinogen, and CRP after PTX treatment [202], and reductions in proteinuria and urinary levels of TNFα and MCP1 after 1 year with add-on PTX to ARB background therapy [203].

To date, the PREDIAN trial is the largest randomized controlled study evaluating the renoprotective effects of PTX in patients with DN under RAS blockade [143]. The study comprised 169 type 2 diabetic subjects with CKD stages 3 or 4. The group of patients that received PTX on top of RAS blockade presented a reduction in the progression of renal disease after two years of follow-up. This reduction was accompanied by a decrease in proteinuria and urinary levels of TNFα. Two meta-analysis also pointed to the reduction of proinflammatory cytokines production as the most likely explanation for the antiproteinuric effect of PTX in patients with DN [144] and concluded that PTX additively reduced proteinuria and TNFα in patients with DN patients receiving RAS inhibitors [145]

PTX inhibits phosphodiesterases (PDEs) activity which, in turn, inactivate the intracellular messenger cyclic adenosine-3,5-monophosphate (cAMP). Therefore, PTX prevents the inactivation of cAMP, resulting in increased levels of cAMP which in turn upregulate protein kinase A (PKA)/cAMP response element-binding (CREB) protein thereby downregulating NF-κB signaling, which causes an anti-inflammatory response via the reduction of IL1, IL6, and TNFα synthesis [146,147]. Moreover, PTX specifically inhibits the isoforms PDE3 and PDE4 which are mainly present in inflammatory cells [204]. In several models of renal disease, PTX is able to attenuate proteinuria via the modulation of signaling pathways or components triggered by inflammatory cytokines [205,206].

Other unexpected renoprotective effect of PTX on DN could come from the stimulation of factors directly related to kidney health. One of these factors is Klotho, a type I single-pass transmembrane protein predominantly expressed in the kidneys in a transmembrane and a soluble form [207]. Many studies demonstrate the antiaging and nephroprotective effects of this protein. Importantly, patients with type 2 diabetes have low soluble Klotho levels [208,209], and biopsies from patients with early stages of DN present diminished renal Klotho expression [210]. These results point to Klotho as a potential early biomarker of renal impairment in type 2 diabetic patients [211]. Interestingly, Klotho has an inverse relationship with inflammation. Thus, proinflammatory cytokines like TNFα and TWEAK (tumor necrosis factor–like weak inducer of apoptosis) exerts a NF-κB-mediated inhibition of renal Klotho expression [212,213]. On the other hand, the addition of Klotho to renal and vascular endothelial cells reduces the synthesis of proinflammatory cytokines [213] and the expression of TNFα-induced adhesion molecules [214]. In the clinical setting, a post-hoc analysis of the PREDIAN trial [148] reported that the administration of PTX reduced serum and urinary TNFα and increased serum and urinary Klotho levels in type 2 diabetic patients with CKD stages 3 and 4. Although the precise mechanisms are unknown, the Klotho stimulatory effect of PTX may come from its anti-inflammatory properties since PTX is able to reduce levels TWEAK, TNFα and albuminuria. Albuminuria causes tubular inflammation and renal injury [215] and also directly reduces the expression of Klotho renal tubular cells [216].

## 4. Conclusions

The burden of global diabetes is predicted to increase dramatically in the coming decades in parallel with the rising of obesity. One of the most important microvascular complications of diabetes is nephropathy, which substantially increases cardiovascular morbidity and mortality, determining a considerable impairment in the quality of this group of patients [217]. Therefore, the need to find therapeutic targets and strategies for treating DN is clearly evident. Conventional treatments provide incomplete protection for the development of renal failure. Recent studies suggest that inflammation is a key factor in the development and progression of DN [218]. Future therapeutic approaches with the ability to modulate inflammatory processes could be useful in the prevention or treatment of DN. These incoming therapies will be focused on the modulation of inflammatory pathways, including targets such as inflammatory cytokines, oxidative stress, JAK/STAT pathway, or NF-κB. Unfortunately, beyond the RAS blockade, there is limited experience regarding the inhibition of inflammatory molecules and pathways in the diabetic milieu. Therefore, further clinical trials are necessary to examine the potential renoprotective efficacy of the modulation of the inflammatory process and to understand how inflammatory pathways interact with other pathogenic factors in the context of DN.

## Figures and Tables

**Table 1:** Main therapies for diabetic kidney disease with anti-inflammatory properties.

Drug	Primary Target	Outcomes	Anti-Inflammatory Effects	Ref.
RAS blockers	Inhibition of ACE or blockade of angiotensin II receptor.	Reduce proteinuria and the progression of nephropathy.	Inhibition of NF-𝜅B, MCP1 gene expression, and macrophage infiltration.	[124]
Methyl bardoxolone	Activation of Nrf2.	Improved renal function in the short- and in the long-term. Stopped by serious adverse events.	Inhibition of NF-𝜅B.	[125]
Emanticap pegol	Blockade of the MCP1 receptor.	Albuminuria reduction compared with baseline, but no significant difference with placebo	Inhibition of MCP1.	[126]
Baricitinib	Inhibition of the JAK/STAT pathway.	Albuminuria reduction in a dose dependent manner.	Reduction of inflammatory biomarkers like ICAM-1, TNFR1 and 2.	[85]
SGLT2 inhibitors	Blockade of glucose reabsorption by SGLT2 at the proximal tubule.	Improved glycemic control. Slower progression of kidney disease and lower rates of clinically relevant renal events.	Reduction of inflammation by targeting the IL-1ß and reduction of hsCRP, TNF𝛼, IL6 and IFN-γ.	[127,128,129,130,131,132,133,134,135]
DPP-4 inhibitors and GLP-1 receptor agonists	Stimulation of glucose-dependent insulin release.	Improved glycemic control and body weight reductions. Renoprotective actions.	Reduction in levels of inflammatory markers.	[136,137,138,139,140,141,142]
Pentoxifylline	Inhibition of phosphodiesterases.	Reduced progression of renal disease and proteinuria.	Downregulation of NF-κB signaling and reduction of inflammatory biomarkers. Increased urinary Klotho.	[143,144,145,146,147,148]

## References

[1] International Diabetes Federation (2017). IDF Diabetes Atlas.

[2] Atkins R.C., Zimmet P. (2010). Diabetic kidney disease: Act now or pay later. Kidney Int..

[3] Ritz E., Rychlík I., Locatelli F., Halimi S. (1999). End-stage renal failure in type 2 diabetes: A medical catastrophe of worldwide dimensions. Am. J. Kidney Dis..

[4] Atkins R.C. (2005). The epidemiology of chronic kidney disease. Kidney Int..

[5] Cooper M.E. (2012). Diabetes: Treating diabetic nephropathy-still an unresolved issue. Nat. Rev. Endocrinol..

[6] Wolf G. (2004). New insights into the pathophysiology of diabetic nephrophathy: From haemodynamics to molecular pathology. Eur. J. Clin. Investig..

[7] Martini S., Eichinger F., Nair V., Kretzler M. (2008). Defining human diabetic nephropathy on themolecular level: Integration of transcriptomic profiles with biological knowledge. Rev. Endocr. Metab. Disord..

[8] Pickup J.C., Crook M.A. (1998). Is Type II diabetes mellitus a disease of the innate immune system?. Diabetologia.

[9] Crook M. (2004). Type 2 diabetes mellitus: A disease of the innate immune system? An update. Diabet. Med..

[10] Pickup J.C. (2004). Inflammation and activated innate immunity in the pathogenesis of type 2 diabetes. Diabetes Care.

[11] Pickup J.C., Mattock M.B., Chusney G.D., Burt D. (1997). NIDDM as a disease of the innate immune system: Association of acute-phase reactants and interleukin-6 with metabolic syndrome X. Diabetologia.

[12] Katsuki A., Sumida Y., Murashima S., Murata K., Takarada Y., Ito K., Fujii M., Tsuchihashi K., Goto H., Nakatani K. (1998). Serum levels of tumor necrosis factor-𝛼 are increased in obese patients with noninsulin-dependent diabetes mellitus. J. Clin. Endocrinol. Metab..

[13] Pickup J.C., Chusney G.D., Thomas S.M., Burt D. (2000). Plasma interleukin-6, tumour necrosis factor 𝛼 and blood cytokine. Life Sci..

[14] Chen J.-W., Gall M.-A., Yokoyama H., Jensen J.S., Deckert M., Parving H.-H. (1996). Raised Serum Sialic Acid Concentration in NIDDM Patients With and Without Diabetic Nephropathy. Diabetes Care.

[15] Rodríguez-Morán M., Guerrero-Romero F. (1999). Increased Levels of C-Reactive Protein in Noncontrolled Type II Diabetic Subjects. J. Diabetes Its Complicat..

[16] Schmidt M.I., Duncan B.B., Sharrett A.R., Lindberg G., Savage P.J., Offenbacher S., Azambuja M.I., Tracy R.P., Heiss G. (1999). Markers of inflammation and prediction of diabetes mellitus in adults (Atherosclerosis Risk in Communities study): A cohort study. Lancet.

[17] Pradhan A.D., Manson J.E., Rifai N., Buring J.E., Ridker P.M. (2001). C-reactive protein, interleukin 6, and risk of developing type 2 diabetes mellitus. JAMA.

[18] Spranger J., Kroke A., Möhlig M., Hoffmann K., Bergmann M.M., Ristow M., Boeing H., Pfeiffer A.F. (2003). Inflammatory cytokines and the risk to develop type 2 diabetes: Results of the prospective population-based European Prospective Investigation into Cancer and Nutrition (EPIC)-Potsdam Study. Diabetes.

[19] Wada J., Makino H. (2013). Inflammation and the pathogenesis of diabetic nephropathy. Clin Sci..

[20] Chow F., Ozols E., Nikolic-Paterson D.J., Atkins R.C., Tesch G.H. (2004). Macrophages in mouse type 2 diabetic nephropathy: Correlation with diabetic state and progressive renal injury. Kidney Int..

[21] Nguyen D., Ping F., Mu W., Hill P., Atkins R.C., Chadban S.J. (2006). Macrophage accumulation in human progressive diabetic nephropathy. Nephrology.

[22] Lim A.K.H., Ma F.Y., Nikolic-Paterson D.J., Kitching A.R., Thomas M.C., Tesch G.H. (2010). Lymphocytes promote albuminuria, but not renal dysfunction or histological damage in a mouse model of diabetic renal injury. Diabetologia.

[23] Moriya R., Manivel J.C., Mauer M. (2004). Juxtaglomerular apparatus T-cell infiltration affects glomerular structure in Type 1 diabetic patients. Diabetologia.

[24] Ferenbach D., Kluth D.C., Hughes J. (2007). Inflammatory Cells in Renal Injury and Repair. Semin. Nephrol..

[25] Bruno G., Merletti F., Biggeri A., Bargero G., Ferrero S., Pagano G., Perin P.C. (2003). Progression to overt nephropathy in type 2 diabetes: The Casale Monferrato Study. Diabetes Care.

[26] Festa A., D’Agostino R., Howard G., Mykkänen L., Tracy R.P., Haffner S.M. (2000). Inflammation and microalbuminuria in nondiabetic and type 2 diabetic subjects: The Insulin Resistance Atherosclerosis Study. Kidney Int..

[27] Tuttle K.R. (2005). Linking metabolism and immunology: Diabetic nephropathy is an inflammatory disease. J. Am. Soc. Nephrol..

[28] Clausen P., Jacobsen P., Rossing K., Jensen J.S., Parving H.-H., Feldt-Rasmussen B. (2000). Plasma concentrations of VCAM-1 and ICAM-1 are elevated in patients with Type 1 diabetes mellitus with microalbuminuria and overt nephropathy. Diabet. Med..

[29] Hojs R., Ekart R., Bevc S., Hojs N. (2016). Markers of Inflammation and Oxidative Stress in the Development and Progression of Renal Disease in Diabetic Patients. Nephron.

[30] Okada S., Shikata K., Matsuda M., Ogawa D., Usui H., Kido Y., Nagase R., Wada J., Shikata Y., Makino H. (2003). Intercellular adhesion molecule-1-deficient mice are resistant against renal injury after induction of diabetes. Diabetes.

[31] Tian S., Chen S.-Y. (2015). Macrophage polarization in kidney diseases. Macrophage.

[32] Klessens C.Q.F., Zandbergen M., Wolterbeek R., Bruijn J.A., Rabelink T.J., Bajema I.M., IJpelaar D.H.T. (2017). Macrophages in diabetic nephropathy in patients with type 2 diabetes. Nephrol. Dial. Transpl..

[33] Liu Y. (2011). Cellular and molecular mechanisms of renal fibrosis. Nat. Rev. Nephrol..

[34] Awad A.S., Kinsey G.R., Khutsishvili K., Gao T., Bolton W.K., Okusa M.D. (2011). Monocyte/macrophage chemokine receptor CCR2 mediates diabetic renal injury. Am. J. Physiol. Physiol..

[35] Chow F.Y., Nikolic-Paterson D.J., Ozols E., Atkins R.C., Tesch G.H. (2005). Intercellular Adhesion Molecule-1 Deficiency Is Protective against Nephropathy in Type 2 Diabetic db/db Mice. J. Am. Soc. Nephrol..

[36] Usui H.K., Shikata K., Sasaki M., Okada S., Matsuda M., Shikata Y., Ogawa D., Kido Y., Nagase R., Yozai K. (2007). Macrophage Scavenger Receptor-A-Deficient Mice Are Resistant Against Diabetic Nephropathy Through Amelioration of Microinflammation. Diabetes.

[37] Devaraj S., Tobias P., Kasinath B.S., Ramsamooj R., Afify A., Jialal I. (2011). Knockout of toll-like receptor-2 attenuates both the proinflammatory state of diabetes and incipient diabetic nephropathy. Arter. Thromb. Vasc. Boil..

[38] Segerer S., Nelson P.J., Schlöndorff D. (2000). Chemokines, chemokine receptors, and renal disease: From basic science to pathophysiologic and therapeutic studies. J. Am. Soc. Nephrol..

[39] Wada T., Furuichi K., Sakai N., Iwata Y., Yoshimoto K., Shimizu M., Takeda S.-I., Takasawa K., Yoshimura M., Kida H. (2000). Up-regulation of monocyte chemoattractant protein-1 in tubulointerstitial lesions of human diabetic nephropathy. Kidney Int..

[40] Banba N., Nakamura T., Matsumura M., Kuroda H., Hattori Y., Kasai K. (2000). Possible relationship of monocyte chemoattractant protein-1 with diabetic nephropathy. Kidney Int..

[41] Aldhahi W., Hamdy O. (2003). Adipokines, inflammation, and the endothelium in diabetes. Curr. Diabetes Rep..

[42] Satirapoj B., Dispan R., Radinahamed P., Kitiyakara C. (2018). Urinary epidermal growth factor, monocyte chemoattractant protein-1 or their ratio as predictors for rapid loss of renal function in type 2 diabetic patients with diabetic kidney disease. BMC Nephrol..

[43] Matoba K., Kawanami D., Tsukamoto M., Kinoshita J., Ito T., Ishizawa S., Kanazawa Y., Yokota T., Murai N., Matsufuji S. (2014). Rho-kinase regulation of TNF-alpha-induced nuclear translocation of NF-kappaB RelA/p65 and M-CSF expression via p38 MAPK in mesangial cells. Am. J. Physiol. Renal Physiol..

[44] Lenda D.M., Kikawada E., Stanley E.R., Kelley V.R. (2003). Reduced macrophage recruitment, proliferation, and activation in colony-stimulating factor-1-deficient mice results in decreased tubular apoptosis during renal inflammation. J. Immunol..

[45] Lim A.K.H., Ma F.Y., Nikolic-Paterson D.J., Thomas M.C., Hurst L.A., Tesch G.H. (2009). Antibody blockade of c-fms suppresses the progression of inflammation and injury in early diabetic nephropathy in obese db/db mice. Diabetologia.

[46] Sugimoto H., Shikata K., Wada J., Horiuchi S., Makino H. (1999). Advanced glycation end products-cytokine-nitric oxide sequence pathway in the development of diabetic nephropathy: Aminoguanidine ameliorates the overexpression of tumour necrosis factor-𝛼 and inducible nitric oxide synthase in diabetic rat glomeruli. Diabetologia.

[47] Jevnikar A.M., Brennan D.C., Singer G.G., Heng J.E., Maslinski W., Wuthrich R.P., Glimcher L.H., Kelley V.E. (1991). Stimulated kidney tubular epithelial cells express membrane associated and secreted TNF𝛼. Kidney Int..

[48] Dong X., Swaminathan S., Bachman L.-A., Croatt A.-J., Nath K.-A., Griffin M.-D. (2007). Resident dendritic cells are the predominant TNF-secreting cell in early renal ischemia–reperfusion injury. Kidney Int..

[49] Noronha I.L., Niemir Z., Stein H., Waldherr R. (1995). Cytokines and growth factors in renal disease. Nephrol. Dial. Transplant..

[50] Mariano F., Bussolati B., Piccoli G., Camussi G. (1997). Renal Vascular Effects of Cytokines. Blood Purif..

[51] Ostendorf T., Burg M., Floege J. (1996). Cytokines and Glomerular Injury. Kidney Blood Press. Res..

[52] Navarro J.F., Mora C., Macıéa M., Garcıéa J. (2003). Inflammatory parameters are independently associated with urinary albumin in type 2 diabetes mellitus. Am. J. Kidney Dis..

[53] Moriwaki Y., Yamamoto T., Shibutani Y., Aoki E., Tsutsumi Z., Takahashi S., Okamura H., Koga M., Fukuchi M., Hada T. (2003). Elevated levels of interleukin-18 and tumor necrosis factor-alpha in serum of patients with type 2 diabetes mellitus: Relationship with diabetic nephropathy. Metabolism.

[54] Navarro J.F., Mora C., Muros M., Garcıa J. (2006). Urinary tumour necrosis factor-alpha excretion independently correlates with clinical markers of glomerular and tubulointerstitial injury in type 2 diabetic patients. Nephrol. Dial. Transpl..

[55] Navarro J.F., Milena F.J., Mora C., Leon C., Garcıa J. (2007). Renal pro-inflammatory cytokine gene expression in diabetic nephropathy: Effect of angiotensin-converting enzyme inhibition and pentoxifylline administration. Am. J. Nephrol..

[56] Navarro J.F., Milena F.J., Mora C., León C., Claverie F., Flores C., García J. (2005). Tumor necrosis factor alpha gene expression in diabetic nephropathy: Relationship with urinary albumin excretion and effect of angiotensin converting enzyme inhibition. Kidney Int..

[57] DiPetrillo K., Gesek F.A. (2004). Pentoxifylline Ameliorates Renal Tumor Necrosis Factor Expression, Sodium Retention, and Renal Hypertrophy in Diabetic Rats. Am. J. Nephrol..

[58] Bertani T., Abbate M., Zoja C., Corna D., Perico N., Ghezzi P., Remuzzi G. (1989). Tumor necrosis factor induces glomerular damage in the rabbit. Am. J. Pathol..

[59] Laster S.M., Wood J.G., Gooding L.R. (1988). Tumor necrosis factor can induce both apoptic and necrotic forms of cell lysis. J. Immunol..

[60] Boyle J.J., Weissberg P.L., Bennett M.R. (2003). Tumor necrosis factor-𝛼 promotes macrophage-induced vascular smooth muscle cell apoptosis by direct and autocrine mechanisms. Arterioscler. Thromb. Vasc. Biol..

[61] Baud L., Perez J., Friedlander G., Ardaillou R. (1988). Tumor necrosis factor stimulates prostaglandin production and cyclic AMP levels in rat cultured mesangial cells. FEBS Lett..

[62] Koike N., Takamura T., Kaneko S. (2007). Induction of reactive oxygen species from isolated rat glomeruli by protein kinase C activation and TNF-𝛼 stimulation, and effects of a phosphodiesterase inhibitor. Life Sci..

[63] McCarthy E.T., Sharma R., Sharma M., Li J.Z., Ge X.L., Dileepan K.N., Savin V.J. (1998). TNF-𝛼 increases albumin permeability of isolated rat glomeruli through the generation of superoxide. J. Am. Soc. Nephrol..

[64] DiPetrillo K., Coutermarsh B., Gesek F.A. (2003). Urinary tumor necrosis factor contributes to sodium retention and renal hypertrophy during diabetes. Am. J. Physiol. Physiol..

[65] Schreiner G.F., Kohan D.E. (1990). Regulation of renal transport processes and hemodynamics by macrophages and lymphocytes. Am. J. Physiol. Physiol..

[66] Yu H.C.M., Burrell L.M., Black M.J., Wu L.L., Dilley R.J., Cooper M.E., Johnston C.I. (1998). Salt induces myocardial and renal fibrosis in normotensive and hypertensive rats. Circulation.

[67] Mahadevan P., Larkins R.G., Fraser J.R., Fosang A.J., Dunlop M.E. (1995). Increased hyaluronan production in the glomeruli from diabetic rats: A link between glucose-induced prostaglandin production and reduced sulphated proteoglycan. Diabetologia.

[68] Suzuki D., Miyazaki M., Naka R., Koji T., Yagame M., Jinde K., Endoh M., Nomoto Y., Sakai H. (1995). In situ hybridization of interleukin 6 in diabetic nephropathy. Diabetes.

[69] Coleman D.L., Ruef C. (1992). Interleukin-6: An autocrine regulator of mesangial cell growth. Kidney Int..

[70] Nosadini R., Velussi M., Brocco E., Bruseghin M., Abaterusso C., Saller A., Vestra M.D., Carraro A., Bortoloso E., Sambataro M. (2000). Course of renal function in type 2 diabetic patients with abnormalities of albumin excretion rate. Diabetes.

[71] Vestra M.D., Mussap M., Gallina P., Bruseghin M., Cernigoi A.M., Saller A., Plebani M., Fioretto P. (2005). Acute-phase markers of inflammation and glomerular structure in patients with type 2 diabetes. J. Am. Soc. Nephrol..

[72] Nakamura A., Shikata K., Hiramatsu M., Nakatou T., Kitamura T., Wada J., Itoshima T., Makino H. (2005). Serum interleukin-18 levels are associated with nephropathy and atherosclerosis in Japanese patients with type 2 diabetes. Diabetes Care.

[73] Wong C.K., Ho A.W.Y., Tong P.C.Y., Yeung C.Y., Kong A.P.S., Lun S.W.M., Chan J.C.N., Lam C.W.K. (2007). Aberrant activation profile of cytokines and mitogen-activated protein kinases in type 2 diabetic patients with nephropathy. Clin. Exp. Immunol..

[74] Okamura H., Tsutsui H., Komatsu T., Yutsudo M., Hakura A., Tanimoto T., Torigoe K., Okura T., Nukada Y., Hattori K. (1995). Cloning of a new cytokine that induces IFN-gamma production by T cells. Nature.

[75] Schwarz M., Wahl M., Resch K., Radeke H.H. (2002). IFN𝛾 induces functional chemokine receptor expression in human mesangial cells. Clin. Exp. Immunol..

[76] Dai S.M., Matsuno H., Nakamura H., Nishioka K., Yudoh K. (2004). Interleukin-18 enhances monocyte tumor necrosis factor 𝛼 and interleukin-1𝛽 production induced by direct contact with T lymphocytes: Implications in rheumatoid arthritis. Arthritis Rheum..

[77] Mariño E., Cardier J.E. (2003). Differential effect of IL-18 on endothelial cell apoptosis mediated by TNF-alpha and Fas (CD95). Cytokine.

[78] Stuyt R.J.L., Netea M.G., Geijtenbeek T.B.H., Kullberg B.J., Dinarello C.A., Van Der Meer J.W.M. (2003). Selective regulation of intercellular adhesion molecule-1 expression by interleukin-18 and interleukin-12 on human monocytes. Immunology.

[79] Fantuzzi G., Reed D.A., Dinarello C.A. (1999). IL-12-induced IFN gamma is dependent on caspase-1 processing of the IL-18 precursor. J. Clin. Investig..

[80] Miyauchi K., Takiyama Y., Honjyo J., Tateno M., Haneda M. (2009). Upregulated IL-18 expression in type 2 diabetic subjects with nephropathy: TGF-beta1 enhanced IL-18 expression in human renal proximal tubular epithelial cells. Diabetes Res. Clin. Pract..

[81] Melnikov V.Y., Ecder T., Fantuzzi G., Siegmund B., Lucia M.S., Dinarello C.A., Schrier R.W., Edelstein C.L. (2001). Impaired IL-18 processing protects caspase-1–deficient mice from ischemic acute renal failure. J. Clin. Investig..

[82] Melnikov V.Y., Faubel S., Siegmund B., Lucia M.S., Ljubanovic D., Edelstein C.L. (2002). Neutrophil-independent mechanisms of caspase-1- and IL-18-mediated ischemic acute tubular necrosis in mice. J. Clin. Investig..

[83] O’Sullivan L.A., Liongue C., Lewis R.S., Stephenson S.E., Ward A.C. (2007). Cytokine receptor signaling through the Jak–Stat–Socs pathway in disease. Mol. Immunol..

[84] Marrero M.B., Banes-Berceli A.K., Stern D.M., Eaton D.C. (2006). Role of the JAK/STAT signaling pathway in diabetic nephropathy. Am. J. Physiol. Physiol..

[85] Tuttle K.R., Brosius F.C., Adler S.G., Kretzler M., Mehta R.L., Tumlin J.A., Tanaka Y., Haneda M., Liu J., Silk M.E. (2018). JAK1/JAK2 inhibition by baricitinib in diabetic kidney disease: Results from a Phase 2 randomized controlled clinical trial. Nephrol. Dial. Transplant..

[86] Li R., Yang N., Zhang L., Huang Y., Zhang R., Wang F., Luo M., Liang Y., Yu X. (2007). Inhibition of JAK/STAT Signaling Ameliorates Mice Experimental Nephrotic Syndrome. Am. J. Nephrol..

[87] Banes-Berceli A.K., Shaw S., Ma G., Brands M., Eaton D.C., Stern D.M., Fulton D., Caldwell R.W., Marrero M.B. (2006). Effect of simvastatin on high glucose- and angiotensin II-induced activation of the JAK/STAT pathway in mesangial cells. Am. J. Physiol. Physiol..

[88] Amiri F., Shaw S., Wang X., Tang J., Waller J.L., Eaton D.C., Marrero M.B. (2002). Angiotensin II activation of the JAK/STAT pathway in mesangial cells is altered by high glucose. Kidney Int..

[89] Berthier C.C., Zhang H., Schin M., Henger A., Nelson R.G., Yee B., Boucherot A., Neusser M.A., Cohen C.D., Carter-Su C. (2009). Enhanced expression of Janus kinase-signal transducer and activator of transcription pathway members in human diabetic nephropathy. Diabetes.

[90] Modesti A., Bertolozzi I., Gamberi T., Marchetta M., Lumachi C., Coppo M., Moroni F., Toscano T., Lucchese G., Gensini G.F. (2005). Hyperglycemia activates JAK2 signaling pathway in human failing myocytes via angiotensin II-mediated oxidative stress. Diabetes.

[91] Choudhury G.G., Ghosh-Choudhury N., Abboud H.E. (1998). Association and direct activation of signal transducer and activator of transcription1alpha by platelet-derived growth factor receptor. J. Clin. Investig..

[92] Banes A.K., Shaw S., Jenkins J., Redd H., Amiri F., Pollock D.M., Marrero M.B. (2004). Angiotensin II blockade prevents hyperglycemia-induced activation of JAK and STAT proteins in diabetic rat kidney glomeruli. Am. J. Physiol. Physiol..

[93] Banes-Berceli A.K.L., Ketsawatsomkron P., Ogbi S., Patel B., Pollock D.M., Marrero M.B. (2007). Angiotensin II and endothelin-1 augment the vascular complications of diabetes via JAK2 activation. Am. J. Physiol. Circ. Physiol..

[94] Ortiz-Muñoz G., Lopez-Parra V., Lopez-Franco O., Fernandez-Vizarra P., Mallavia B., Flores C., Sanz A., Blanco J., Mezzano S., Ortiz A. (2010). Suppressors of cytokine signaling abrogate diabetic nephropathy. J. Am. Soc. Nephrol..

[95] Guijarro C., Egido J. (2001). Transcription factor-kappa B (NF-kappa B) and renal disease. Kidney Int..

[96] Navarro-Gonzalez J.F., Mora-Fernández C., De Fuentes M.M., García-Pérez J. (2011). Inflammatory molecules and pathways in the pathogenesis of diabetic nephropathy. Nat. Rev. Nephrol..

[97] Pérez-Morales R.E., Del Pino M.D., Valdivielso J.M., Ortiz A., Mora-Fernández C., Navarro-González J.F. (2018). Inflammation in Diabetic Kidney Disease. Nephron.

[98] Pieper G.M., Haq R.U. (1997). Activation of nuclear factor-kappaB in cultured endothelial cells by increased glucose concentration: Prevention by calphostin C. J. Cardiovasc. Pharmacol..

[99] Mezzano S., Aros C., Droguett A., Burgos M.E., Ardiles L., Flores C., Schneider H., Ruiz-Ortega M., Egido J. (2004). NF-kappaB activation and overexpression of regulated genes in human diabetic nephropathy. Nephrol. Dial. Transplant..

[100] Han S.-Y., Kim C.-H., Kim H.-S., Jee Y.-H., Song H.-K., Lee M.-H., Han K.-H., Kim H.-K., Kang Y.-S., Han J.-Y. (2006). Spironolactone Prevents Diabetic Nephropathy through an Anti-Inflammatory Mechanism in Type 2 Diabetic Rats. J. Am. Soc. Nephrol..

[101] Ohga S., Shikata K., Yozai K., Okada S., Ogawa D., Usui H., Wada J., Shikata Y., Makino H. (2007). Thiazolidinedione ameliorates renal injury in experimental diabetic rats through anti-inflammatory effects mediated by inhibition of NF-kappaB activation. Am. J. Physiol. Renal Physiol..

[102] Ko G.J., Kang Y.S., Han S.Y., Lee M.H., Song H.K., Han K.H., Kim H.K., Han J.Y., Cha D.R. (2008). Pioglitazone attenuates diabetic nephropathy through an anti-inflammatory mechanism in type 2 diabetic rats. Nephrol. Dial. Transplant..

[103] Zhang Z., Yuan W., Sun L., Szeto F.L., Wong K.E., Li X., Kong J., Li Y.C. (2007). 1,25-Dihydroxyvitamin D3 targeting of NF-kappaB suppresses high glucose-induced MCP-1 expression in mesangial cells. Kidney Int..

[104] Lee W.C., Chen H.C., Wang C.Y., Lin P.Y., Ou T.T., Chen C.C., Wen M.C., Wang J., Lee H.J. (2010). Cilostazol ameliorates nephropathy in type 1 diabetic rats involving improvement in oxidative stress and regulation of TGF-Beta and NF-kappaB. Biosci. Biotechnol. Biochem..

[105] Soetikno V., Sari F.R., Veeraveedu P.T., Thandavarayan R.A., Harima M., Sukumaran V., Lakshmanan A.P., Suzuki K., Kawachi H., Watanabe K. (2011). Curcumin ameliorates macrophage infiltration by inhibiting NF-κB activation and proinflammatory cytokines in streptozotocin induced-diabetic nephropathy. Nutr. Metab. (Lond).

[106] Matoba K., Kawanami D., Okada R., Tsukamoto M., Kinoshita J., Ito T., Ishizawa S., Kanazawa Y., Yokota T., Murai N. (2013). Rho-kinase inhibition prevents the progression of diabetic nephropathy by downregulating hypoxia-inducible factor 1alpha. Kidney Int..

[107] Kawanami D., Matoba K., Utsunomiya K. (2016). Signaling pathways in diabetic nephropathy. Histol. Histopathol..

[108] Kawanami D., Matoba K., Kanazawa Y., Ishizawa S., Yokota T., Utsunomiya K. (2011). Thrombin induces MCP-1 expression through Rho-kinase and subsequent p38MAPK/NF-kappaB signaling pathway activation in vascular endothelial cells. Biochem. Biophys. Res. Commun..

[109] Shimada H., Rajagopalan L.E. (2010). Rho kinase-2 activation in human endothelial cells drives lysophosphatidic acid-mediated expression of cell adhesion molecules via NF-kappaB p65. J. Biol. Chem..

[110] Zhao D., Kuhnt-Moore S., Zeng H., Wu J.S., Moyer M.P., Pothoulakis C. (2003). Neurotensin stimulates IL-8 expression in human colonic epithelial cells through Rho GTPase-mediated NF-kappa B pathways. Am. J. Physiol. Cell Physiol..

[111] Meyer-Schwesinger C., Dehde S., von Ruffer C., Gatzemeier S., Klug P., Wenzel U.O., Stahl R.A., Thaiss F., Meyer T.N. (2009). Rho kinase inhibition attenuates LPS-induced renal failure in mice in part by attenuation of NF-kappaB p65 signaling. Am. J. Physiol. Renal Physiol..

[112] Kobayashi E.H., Suzuki T., Funayama R., Nagashima T., Hayashi M., Sekine H., Tanaka N., Moriguchi T., Motohashi H., Nakayama K. (2016). Nrf2 suppresses macrophage inflammatory response by blocking proinflammatory cytokine transcription. Nat. Commun..

[113] Lazaro I., Lopez-Sanz L., Bernal S., Oguiza A., Recio C., Melgar A., Jimenez-Castilla L., Egido J., Madrigal-Matute J., Gomez-Guerrero C. (2018). Nrf2 Activation Provides Atheroprotection in Diabetic Mice Through Concerted Upregulation of Antioxidant, Anti-inflammatory, and Autophagy Mechanisms. Front. Pharmacol..

[114] Zheng H., Whitman S.A., Wu W., Wondrak G.T., Wong P.K., Fang D., Zhang D.D. (2011). Therapeutic potential of Nrf2 activators in streptozotocin-induced diabetic nephropathy. Diabetes..

[115] American Diabetes Association (2019). 6. Glycemic targets: Standards of medical care in diabetes—2019. Diabetes Care.

[116] Ruggenenti P., Cravedi P., Remuzzi G. (2010). The RAAS in the pathogenesis and treatment of diabetic nephropathy. Nat. Rev..

[117] American Diabetes Association (2019). 11. Microvascular complications and foot care: Standards of medical care in diabetes—2019. Diabetes Care.

[118] Parving H.H., Brenner B.M., McMurray J.J., de Zeeuw D., Haffner S.M., Solomon S.D., Chaturvedi N., Persson F., Desai A.S., Nicolaides M. (2012). Cardiorenal end points in a trial of aliskiren for type 2 diabetes. N. Engl. J. Med..

[119] Fried L.F., Emanuele N., Zhang J.H., Brophy M., Conner T.A., Duckworth W., Leehey D.J., McCullough P.A., O’Connor T., Palevsky P.M. (2013). Combined angiotensin inhibition for the treatment of diabetic nephropathy. N. Engl. J. Med..

[120] Packham D.K., Wolfe R., Reutens A.T., Berl T., Heerspink H.L., Rohde R., Ivory S., Lewis J., Raz I., Wiegmann T.B. (2012). Collaborative Study Group. Sulodexide fails to demonstrate renoprotection in overt type 2 diabetic nephropathy. J. Am. Soc. Nephrol..

[121] Lewis E.J., Greene T., Spitalewiz S., Blumenthal S., Berl T., Hunsicker L.G., Pohl M.A., Rohde R.D., Raz I., Yerushalmy Y. (2012). Collaborative Study Group. Pyridorin in type 2 diabetic nephropathy. J. Am. Soc. Nephrol..

[122] Mann J.F., Green D., Jamerson K., Ruilope L.M., Kuranoff S.J., Littke T., Viberti G. (2010). ASCEND Study Group. Avosentan for overt diabetic nephropathy. J. Am. Soc. Nephrol..

[123] de Zeeuw D., Akizawa T., Audhya P., Bakris G.L., Chin M., Christ-Schmidt H., Goldsberry A., Houser M., Krauth M., Lambers Heerspink H.J. (2013). BEACON Trial Investigators. Bardoxolone methyl in type 2 diabetes and stage 4 chronic kidney disease. N. Engl. J. Med..

[124] Lee F.T.H., Cao Z., Long D.M., Panagiotopoulos S., Jerums G., Cooper M.E., Forbes J.M. (2004). Interactions between angiotensin II and NF-𝜅B-dependent pathways in modulating macrophage infiltration in experimental diabetic nephropathy. J. Am. Soc. Nephrol..

[125] Dinkova-Kostova A.T., Liby K.T., Stephenson K.K., Holtzclaw W.D., Gao X., Suh N., Williams C., Risingsong R., Honda T., Gribble G.W. (2005). Extremely potent triterpenoid inducers of the phase 2 response: Correlations of protection against oxidant and inflammatory stress. Proc. Natl. Acad. Sci. USA.

[126] Menne J., Eulberg D., Beyer D., Baumann M., Saudek F., Valkusz Z., Więcek A., Haller H., Emapticap Study Group (2017). C-C motif-ligand 2 inhibition with emapticap pegol (NOX-E36) in type 2 diabetic patients with albuminuria. Nephrol. Dial. Transplant..

[127] Ridker P.M., Everett B.M., Thuren T., MacFadyen J.G., Chang W.H., Ballantyne C., Fonseca F., Nicolau J., Koenig W., Anker S.D. (2017). Antiinflammatory Therapy with Canakinumab for Atherosclerotic Disease. N. Engl. J. Med..

[128] Ridker P.M., MacFadyen J.G., Everett B.M., Libby P., Thuren T., Glynn R.J., Kastelein J., Koenig W., Genest J., Lorenzatti A. (2018). Relationship of C-reactive protein reduction to cardiovascular event reduction following treatment with canakinumab: A secondary analysis from the CANTOS randomised controlled trial. Lancet.

[129] Matsumura M., Nakatani Y., Tanka S., Aoki C., Sagara M., Yanagi K., Suzuki K., Aso Y. (2017). Efficacy of Additional Canagliflozin Administration to Type 2 Diabetes Patients Receiving Insulin Therapy: Examination of Diurnal Glycemic Patterns Using Continuous Glucose Monitoring (CGM). Diabetes Ther..

[130] Garvey W.T., Van Gaal L., Leiter L.A., Vijapurkar U., List J., Cuddihy R., Ren J., Davies M.J. (2018). Effects of canagliflozin versus glimepiride on adipokines and inflammatory biomarkers in type 2 diabetes. Metabolism.

[131] Ferrannini E., Ramos S.J., Salsali A., Tang W., List J.F. (2010). Dapagliflozin monotherapy in type 2 diabetic patients with inadequate glycemic control by diet and exercise: A randomized, double-blind, placebo-controlled, phase 3 trial. Diabetes Care.

[132] Okamoto A., Yokokawa H., Sanada H., Naito T. (2016). Changes in Levels of Biomarkers Associated with Adipocyte Function and Insulin and Glucagon Kinetics during Treatment with Dapagliflozin Among Obese Type 2 Diabetes Mellitus Patients. Drugs R&D.

[133] Hattori S. (2018). Empagliflozin decreases remnant-like particle cholesterol in type 2 diabetes patients with insulin resistance. J. Diabetes Investig..

[134] Tobita H., Sato S., Miyake T., Ishihara S., Kinoshita Y. (2017). Effects of Dapagliflozin on Body Composition and Liver Tests in Patients with Nonalcoholic Steatohepatitis Associated with Type 2 Diabetes Mellitus: A Prospective, Open-label, Uncontrolled Study. Curr. Ther. Res..

[135] Sato T., Aizawa Y., Yuasa S., Kishi S., Fuse K., Fujita S., Ikeda Y., Kitazawa H., Takahashi M., Sato M. (2018). The effect of dapagliflozin treatment on epicardial adipose tissue volume. Cardiovasc. Diabetol..

[136] Kröller-Schön S., Knorr M., Hausding M., Oelze M., Schuff A., Schell R., Sudowe S., Scholz A., Daub S., Karbach S. (2012). Glucose-independent improvement of vascular dysfunction in experimental sepsis by dipeptidyl-peptidase 4 inhibition. Cardiovasc. Res..

[137] Kanasaki K. (2018). The role of renal dipeptidyl peptidase-4 in kidney disease: Renal effects of dipeptidyl peptidase-4 inhibitors with a focus on linagliptin. Clin. Sci..

[138] Barbieri M., Rizzo M.R., Marfella R., Boccardi V., Esposito A., Pansini A., Paolisso G. (2013). Decreased carotid atherosclerotic process by control of daily acute glucose fluctuations in diabetic patients treated by DPP-IV inhibitors. Atheroscleosis.

[139] Kawanami D., Matoba K., Sango K., Utsunomiya K. (2016). Incretin-Based Therapies for Diabetic Complications: Basic Mechanisms and Clinical Evidence. Int. J. Mol. Sci..

[140] Kodera R., Shikata K., Kataoka H.U., Takatsuka T., Miyamoto S., Sasaki M., Kajitani N., Nishishita S., Sarai K., Hirota D. (2011). Glucagon-like peptide-1 receptor agonist ameliorates renal injury through its anti-inflammatory action without lowering blood glucose level in a rat model of type 1 diabetes. Diabetologia.

[141] Jo C.H., Kim S., Park J.-S., Kim G.-H. (2018). Anti-Inflammatory Action of Sitagliptin and Linagliptin in Doxorubicin Nephropathy. Kidney Blood Press. Res..

[142] Ferdinand K.C., White W.B., Calhoun D.A., Lonn E.M., Sager P.T., Brunelle R., Jiang H.H., Threlkeld R.J., Robertson K.E., Geiger M.J. (2014). Effects of the Once-Weekly Glucagon-Like Peptide-1 Receptor Agonist Dulaglutide on Ambulatory Blood Pressure and Heart Rate in Patients With Type 2 Diabetes Mellitus. Hypertension.

[143] Navarro-González J.F., Mora-Fernández C., Muros de Fuentes M., Chahin J., Méndez M.L., Gallego E., Macía M., del Castillo N., Rivero A., Getino M.A. (2015). Effect of pentoxifylline on renal function and urinary albumin excretion in patients with diabetic kidney disease: The PREDIAN trial. J. Am. Soc. Nephrol..

[144] McCormick B.B., Sydor A., Akbari A., Fergusson D., Doucette S., Knoll G. (2008). The Effect of Pentoxifylline on Proteinuria in Diabetic Kidney Disease: A Meta-analysis. Am. J. Kidney Dis..

[145] Tian M.-L., Shen Y., Sun Z.-L., Zha Y. (2015). Efficacy and safety of combining pentoxifylline with angiotensin-converting enzyme inhibitor or angiotensin II receptor blocker in diabetic nephropathy: A meta-analysis. Int. Urol. Nephrol..

[146] Cheng J., Grande J.P. (2007). Cyclic nucleotide phosphodiesterase (PDE) inhibitors: Novel therapeutic agents for progressive renal disease. Exp. Boil. Med..

[147] Ward A., Clissold S.P. (1987). Pentoxifylline. A review of its pharmacodynamic and pharmacokinetic properties, and its therapeutic efficacy. Drugs.

[148] Navarro-González J.F., Sanchez-Niño M.D., Donate-Correa J., Martin-Nuñez E., Ferri C., Pérez-Delgado N., Gorriz J.L., Martínez-Castelao A., Ortiz A., Mora-Fernández C. (2018). Effects of Pentoxifylline on Soluble Klotho Concentrations and Renal Tubular Cell Expression in Diabetic Kidney Disease. Diabetes Care.

[149] Hostetter T.H. (2001). Prevention of end-stage renal disease due to type 2 diabetes. N. Engl. J. Med..

[150] Moreno J.A., Gomez-Guerrero C., Mas S., Sanz A.B., Lorenzo O., Ruiz-Ortega M., Opazo L., Mezzano S., Egido J. (2018). Targeting inflammation in diabetic nephropathy: A tale of hope. Expert. Opin. Investig. Drugs.

[151] Sanz A.B., Sanchez-Niño M.D., Ramos A.M., Moreno J.A., Santamaria B., Ruiz-Ortega M., Egido J., Ortiz A. (2010). NF-kappaB in renal inflammation. J. Am. Soc. Nephrol..

[152] Pergola P.E., Raskin P., Toto R.D., Meyer C.J., Huff J.W., Grossman E.B., Krauth M., Ruiz S., Audhya P., Christ-Schmidt H. (2011). Bardoxolone methyl and kidney function in CKD with type 2 diabetes. N. Engl. J. Med..

[153] Dagenais N.J., Jamali F. (2005). Protective effects of angiotensin II interruption: Evidence for antiinflammatory actions. Pharmacotherapy.

[154] Dandona P., Dhindsa S., Ghanim H., Chaudhuri A. (2007). Angiotensin II and inflammation: The effect of angiotensinconverting enzyme inhibition and angiotensin II receptor blockade. J. Hum. Hypertens..

[155] Han J., Thompson P., Beutler B. (1990). Dexamethasone and pentoxifylline inhibit endotoxin-induced cachectin/tumor necrosis factor synthesis at separate points in the signaling pathway. J. Exp. Med..

[156] Takebayashi K., Matsumoto S., Aso Y., Inukai T. (2006). Aldosterone blockade attenuates urinary monocyte chemoattractant protein-1 and oxidative stress in patients with type 2 diabetes complicated by diabetic nephropathy. J. Clin. Endocrinol. Metab..

[157] Ninichuk V., Clauss S., Kulkarni O., Schmid H., Segerer S., Radomska E., Eulberg D., Buchner K., Selve N., Klussmann S. (2008). Late onset of Ccl2 blockade with the Spiegelmer mNOX-E36-30 PEG prevents glomerulosclerosis and improves glomerular filtration rate in db/db mice. Am. J. Pathol..

[158] Sayyed S.G., Ryu M., Kulkarni O.P., Schmid H., Lichtnekert J., Grüner S., Green L., Mattei P., Hartmann G., Anders H.J. (2011). An orally active chemokine receptor CCR2 antagonist prevents glomerulosclerosis and renal failure in type 2 diabetes. Kidney Int..

[159] Sullivan T., Miao Z., Dairaghi D.J., Krasinski A., Wang Y., Zhao B.N., Baumgart T., Ertl L.S., Pennell A., Seitz L. (2013). CCR2 antagonist CCX140-B provides renal and glycemic benefits in diabetic transgenic human CCR2 knockin mice. Am. J. Physiol. Renal Physiol..

[160] Seok S.J., Lee E.S., Kim G.T., Hyun M., Lee J.H., Chen S., Choi R., Kim H.M., Lee E.Y., Chung C.H. (2013). Blockade of CCL2/CCR2 signalling ameliorates diabetic nephropathy in db/db mice. Nephrol. Dial. Transplant..

[161] Toth-Manikowski S., Atta M.G. (2015). Diabetic Kidney Disease: Pathophysiology and Therapeutic Targets. J. Diabetes Res..

[162] Fernandez-Fernandez B., Ortiz A., Gomez-Guerrero C., Egido J. (2014). Therapeutic approaches to diabetic nephropathy—Beyond the RAS. Nat. Rev. Nephrol..

[163] Hsu J.D., Wu C.C., Hung C.N., Wang C.J., Huang H.P. (2016). Myrciaria cauliflora extract improves diabetic nephropathy via suppression of oxidative stress and inflammation in streptozotocin-nicotinamide mice. J. Food Drug. Anal..

[164] Li P., Chen Y., Liu J., Hong J., Deng Y., Yang F., Jin X., Gao J., Li J., Fang H. (2015). Efficacy and safety of tangshen formula on patients with type 2 diabetic kidney disease: A multicenter double-blinded randomized placebo-controlled trial. PLoS ONE.

[165] Ghosh R.K., Ghosh S.M., Chawla S., Jasdanwala S.A. (2012). SGLT2 inhibitors: A new emerging therapeutic class in the treatment of type 2 diabetes mellitus. J. Clin. Pharmacol..

[166] Thomas M.C., Cherney D.Z.I. (2018). The actions of SGLT2 inhibitors on metabolism, renal function and blood pressure. Diabetologia.

[167] Wanner C., Inzucchi S.E., Lachin J.M., Fitchett D., von Eynatten M., Mattheus M., Johansen O.E., Woerle H.J., Broedl U.C., Zinman B. (2016). Empagliflozin and Progression of Kidney Disease in Type 2 Diabetes. N. Engl. J. Med..

[168] Perkovic V., de Zeeuw D., Mahaffey K.W., Fulcher G., Erondu N., Shaw W., Barrett T.D., Weidner-Wells M., Deng H., Matthews D.R. (2018). Canagliflozin and renal outcomes in type 2 diabetes: Results from the CANVAS Program randomised clinical trials. Lancet Diabetes Endocrinol..

[169] Perkovic V., Jardine M.J., Neal B., Bompoint S., Heerspink H.J.L., Charytan D.M., Edwards R., Agarwal R., Bakris G., Bull S. (2019). Canagliflozin and Renal Outcomes in Type 2 Diabetes and Nephropathy. N. Engl. J. Med..

[170] Scheen A.J. (2016). Reduction in cardiovascular and all-cause mortality in the EMPA-REG OUTCOME trial: A critical analysis. Diabetes Metab..

[171] Staels B. (2017). Cardiovascular protection by sodium-glucose cotransporter 2 inhibitors: Potential mechanisms. Am. J. Cardiol..

[172] Vallon V., Thomson S.C. (2017). Targeting renal glucose reabsorption to treat hyperglycaemia: The pleiotropic effects of SGLT2 inhibition. Diabetologia.

[173] Sharma A., Tate M., Mathew G., Vince J.E., Ritchie R.H., De Haan J.B. (2018). Oxidative Stress and NLRP3-Inflammasome Activity as Significant Drivers of Diabetic Cardiovascular Complications: Therapeutic Implications. Front. Physiol..

[174] Ishibashi Y., Matsui T., Yamagishi S. (2016). Tofogliflozin, a highly selective inhibitor of SGLT2 blocks proinflammatory and proapoptotic effects of glucose overload on proximal tubular cells partly by suppressing oxidative stress generation. Horm. Metab. Res..

[175] Jaikumkao K., Pongchaidecha A., Chueakula N., Thongnak L.O., Wanchai K., Chatsudthipong V., Chattipakorn N., Lungkaphin A. (2018). Dapagliflozin, a SGLT2 inhibitor, slows the progression of renal complications through the suppression of renal inflammation, ER stress, and apoptosis in pre-diabetic rats. Diabetes Obes. Metab..

[176] Ishibashi Y., Matsui T., Yamagishi S.-I. (2016). Tofogliflozin, a selective inhibitor of sodium-glucose cotransporter 2, suppresses renal damage in KKAy/Ta mice, obese and type 2 diabetic animals. Diabetes Vasc. Dis. Res..

[177] Panchapakesan U., Pegg K., Gross S., Komala M.G., Mudaliar H., Forbes J., Pollock C., Mather A. (2013). Effects of SGLT2 inhibition in human kidney proximal tubular cells—Renoprotection in diabetic nephropathy?. PLoS ONE.

[178] Han J.H., Oh T.J., Lee G., Maeng H.J., Lee D.H., Kim K.M., Choi S.H., Jang H.C., Lee H.S., Park K.S. (2017). The beneficial effects of empagliflozin, an SGLT2 inhibitor, on atherosclerosis in ApoE -/- mice fed a western diet. Diabetologia.

[179] Vallon V., Gerasimova M., Rose M.A., Masuda T., Satriano J., Mayoux E., Koepsell H., Thomson S.C., Rieg T. (2014). SGLT2 inhibitor empagliflozin reduces renal growth and albuminuria in proportion to hyperglycemia and prevents glomerular hyperfiltration in diabetic Akitamice. Am. J. Physiol. Renal Physiol..

[180] Lin B., Koibuchi N., Hasegawa Y., Sueta D., Toyama K., Uekawa K., Ma M., Nakagawa T., Kusaka H., Kim-Mitsuyama S. (2014). Glycemic control with empagliflozin, a novel selective SGLT2 inhibitor, ameliorates cardiovascular injury and cognitive dysfunction in obese and type 2 diabetic mice. Cardiovasc. Diabetol..

[181] Terami N., Ogawa D., Tachibana H., Hatanaka T., Wada J., Nakatsuka A., Eguchi J., Horiguchi C.S., Nishii N., Yamada H. (2014). Long-Term Treatment with the Sodium Glucose Cotransporter 2 Inhibitor, Dapagliflozin, Ameliorates Glucose Homeostasis and Diabetic Nephropathy in db/db Mice. PLoS ONE.

[182] Hattori S. (2011). Sitagliptin reduces albuminuria in patients with type 2 diabetes [Rapid Communication]. Endocr. J..

[183] Sakata K., Hayakawa M., Yano Y., Tamaki N., Yokota N., Eto T., Watanabe R., Hirayama N., Matsuo T., Kuroki K. (2013). Efficacy of alogliptin, a dipeptidyl peptidase-4 inhibitor, on glucose parameters, the activity of the advanced glycation end product (AGE)—Receptor for AGE (RAGE) axis and albuminuria in Japanese type 2 diabetes. Diabetes/Metab. Res. Rev..

[184] US Food & Drug Administration Drugs-FDA: FDA Approved Drug Products. www.accessdata.fda.gov.

[185] Blagosklonnaia I.V., Mamedov R., Kozlov V.V., Emanuél’ V.L., Kudriashova M.I. (1982). [Effect of trental on indices kidney function in diabetes mellitus]. Probl. Endocrinol..

[186] Sinzinger H. (1983). Pentoxifylline enhances formation of prostacyclin from rat vascular and renal tissue. Prostaglandins Leukot. Med..

[187] Doherty G.M., Jensen J.C., Alexander H.R., Buresh C.M., Norton J.A. (1991). Pentoxifylline suppression of tumor necrosis factor gene transcription. Surgery.

[188] Voisin L., Breuillé D., Ruot B., Rallière C., Rambourdin F., Dalle M., Obled C. (1998). Cytokine modulation by PX differently affects specific acute phase proteins during sepsis in rats. Am. J. Physiol. Content.

[189] Strutz F., Heeg M., Kochsiek T., Siemers G., Zeisberg M., Müller G.A. (2000). Effects of pentoxifylline, pentifylline and gamma-interferon on proliferation, differentiation, and matrix synthesis of human renal fibroblasts. Nephrol. Dial. Transplant..

[190] Abdel-Salam O. (2003). The anti-inflammatory effects of the phosphodiesterase inhibitor pentoxifylline in the rat. Pharmacol. Res..

[191] Dávila-Esqueda M.E., Martínez-Morales F. (2004). Pentoxifylline Diminishes the Oxidative Damage to Renal Tissue Induced by Streptozotocin in the Rat. Exp. Diabesity Res..

[192] Navarro-González J.F., Mora-Fernández C. (2008). The Role of Inflammatory Cytokines in Diabetic Nephropathy. J. Am. Soc. Nephrol..

[193] Donate-Correa J., Martin-Nuñez E., Muros-De-Fuentes M., Mora-Fernández C., Navarro-Gonzalez J.F. (2015). Inflammatory Cytokines in Diabetic Nephropathy. J. Diabetes Res..

[194] Cooper A. (2004). Pentoxifylline Improves Hemoglobin Levels in Patients with Erythropoietin-resistant Anemia in Renal Failure. J. Am. Soc. Nephrol..

[195] Fernandes J.L., De Oliveira R.T.D., Mamoni R.L., Coelho O.R., Nicolau J.C., Blotta M.H.S., Serrano C.V. (2008). Pentoxifylline reduces pro-inflammatory and increases anti-inflammatory activity in patients with coronary artery disease—A randomized placebo-controlled study. Atherosclerosis.

[196] Mohammadpour A.H., Falsoleiman H., Shamsara J., Abadi G.A., Rasooli R., Ramezani M. (2014). Pentoxifylline Decreases Serum Level of Adhesion Molecules in Atherosclerosis Patients. Iran. Biomed. J..

[197] Navarro J.F., Mora C., Rivero A., Gallego E., Chahin J., Macía M., Méndez M.L., García J. (1999). Urinary protein excretion and serum tumor necrosis factor in diabetic patients with advanced renal failure: Effects of pentoxifylline administration. Am. J. Kidney Dis..

[198] Aminorroaya A., Janghorbani M., Rezvanian H., Aminian T., Gharavi M., Amini M. (2005). Comparison of the Effect of Pentoxifylline and Captopril on Proteinuria in Patients with Type 2 Diabetes mellitus. Nephron Clin. Pr..

[199] Rodríguez-Morán M., Guerrero-Romero F. (2005). Pentoxifylline is as effective as captopril in the reduction of microalbuminuria in non-hypertensive type 2 diabetic patients—A randomized, equivalent trial. Clin. Nephrol..

[200] Navarro J.F., Mora C., Muros M., García J. (2005). Additive Antiproteinuric Effect of Pentoxifylline in Patients with Type 2 Diabetes under Angiotensin II Receptor Blockade: A Short-Term, Randomized, Controlled Trial. J. Am. Soc. Nephrol..

[201] Rodríguez-Morán M., González-González G., Bermúdez-Barba M., De La Garza C.M., Tamez-Pérez H., Martínez-Martínez F., Guerrero-Romero F. (2006). Effects of pentoxifylline on the urinary protein excretion profile of type 2 diabetic patients with microproteinuria—A double-blind, placebo-controlled randomized trial. Clin. Nephrol..

[202] Goicoechea M., De Vinuesa S.G., Quiroga B., Verdalles U., Barraca D., Yuste C., Panizo N., Verde E., Muñoz M.A., Luño J. (2012). Effects of pentoxifylline on inflammatory parameters in chronic kidney disease patients: A randomized trial. J. Nephrol..

[203] Lin S.-L., Chen Y.-M., Chiang W.-C., Wu K.-D., Tsai T.-J. (2008). Effect of Pentoxifylline in Addition to Losartan on Proteinuria and GFR in CKD: A 12-Month Randomized Trial. Am. J. Kidney Dis..

[204] Tenor H., Schudt C., Schudt C., Dent G., Rabe K.F. (1996). Analysis of PDE Isoenzyme Profiles in Cells and Tissues by Pharmacological Methods. Phosphodiesterase Inhibitors.

[205] Chen Y.M., Ng Y.Y., Lin S.L., Chiang W.C., Lan H.Y., Tsai T.J. (2004). Pentoxifylline suppresses renal tumor necrosis factor-alpha and ameliorates experimental crescentic glomerulonephritis in rats. Nephrol. Dial. Transplant..

[206] Garcia F.A.D.O., Rebouças J.F., Balbino T.Q., Da Silva T.G., De Carvalho-Júnior C.H.R., Cerqueira G.S., Brito G.A.D.C., Viana G.S.D.B. (2015). Pentoxifylline reduces the inflammatory process in diabetic rats: Relationship with decreases of pro-inflammatory cytokines and inducible nitric oxide synthase. J. Inflamm..

[207] Matsumura Y., Aizawa H., Shiraki-Iida T., Nagai R., Kuro O.M., Nabeshima Y.-I. (1998). Identification of the human klotho gene and its two transcripts encoding membrane and secreted klotho protein. Biochem. Biophys. Res. Commun..

[208] Liu J.-J., Liu S., Morgenthaler N.G., Wong M.D., Tavintharan S., Sum C.F., Lim S.C. (2014). Association of plasma soluble α-klotho with pro-endothelin-1 in patients with type 2 diabetes. Atherosclerosis.

[209] Wu C., Wang Q., Lv C., Qin N., Lei S., Yuan Q., Wang G. (2014). The changes of serum sKlotho and NGAL levels and their correlation in type 2 diabetes mellitus patients with different stages of urinary albumin. Diabetes Res. Clin. Pr..

[210] Asai O., Nakatani K., Tanaka T., Sakan H., Imura A., Yoshimoto S., Samejima K.-I., Yamaguchi Y., Matsui M., Akai Y. (2012). Decreased renal α-Klotho expression in early diabetic nephropathy in humans and mice and its possible role in urinary calcium excretion. Kidney Int..

[211] Kim S.S., Song S.H., Kim I.J., Lee E.Y., Lee S.M., Chung C.H., Kwak I.S., Lee E.K., Kim Y.K. (2016). Decreased plasma α-Klotho predict progression of nephropathy with type 2 diabetic patients. J. Diabetes Complicat..

[212] Moreno J.A., Izquierdo M.C., Sanchez-Niño M.D., Suárez-Alvarez B., Lopez-Larrea C., Jakubowski A., Blanco J., Ramírez R., Selgas R., Ruiz-Ortega M. (2011). The inflammatory cytokines TWEAK and TNFα reduce renal klotho expression through NF-κB. J. Am. Soc. Nephrol..

[213] Zhao Y., Banerjee S., Dey N., Lejeune W.S., Sarkar P.S., Brobey R., Rosenblatt K.P., Tilton R.G., Choudhary S. (2011). Klotho Depletion Contributes to Increased Inflammation in Kidney of the db/db Mouse Model of Diabetes via RelA (Serine)536 Phosphorylation. Diabetes.

[214] Maekawa Y., Ishikawa K., Yasuda O., Oguro R., Hanasaki H., Kida I., Takemura Y., Ohishi M., Katsuya T., Rakugi H. (2009). Klotho suppresses TNF-alpha-induced expression of adhesion molecules in the endothelium and attenuates NF-kappaB activation. Endocrine.

[215] Jheng H.-F., Tsai P.-J., Chuang Y.-L., Shen Y.-T., Tai T.-A., Chen W.-C., Chou C.-K., Ho L.-C., Tang M.-J., Lai K.-T.A. (2015). Albumin stimulates renal tubular inflammation through an HSP70-TLR4 axis in mice with early diabetic nephropathy. Dis. Model. Mech..

[216] Fernandez-Fernandez B., Izquierdo M.C., Valiño-Rivas L., Nastou D., Sanz A.B., Ortiz A., Sanchez-Niño M.D. (2018). Albumin downregulates Klotho in tubular cells. Nephrol. Dial. Transplant..

[217] Canto E.D., Ceriello A., Rydén L., Ferrini M., Hansen T.B., Schnell O., Standl E., Beulens J.W. (2019). Diabetes as a cardiovascular risk factor: An overview of global trends of macro and micro vascular complications. Eur. J. Prev. Cardiol..

[218] Shahzad K., Bock F., Dong W., Wang H., Kopf S., Kohli S., Al-Dabet M.M., Ranjan S., Wolter J., Wacker C. (2015). Nlrp3-inflammasome activation in non-myeloid-derived cells aggravates diabetic nephropathy. Kidney Int..

